# The Potential Role of *Brassica napus* Metallothioneins in Salt Stress and Interactions with Plant Growth-Promoting Bacteria

**DOI:** 10.3390/genes16020166

**Published:** 2025-01-28

**Authors:** Agnieszka Mierek-Adamska, Wioleta Tylman-Mojżeszek, Agnieszka Pawełek, Milena Kulasek, Grażyna B. Dąbrowska

**Affiliations:** Department of Genetics, Faculty of Biological and Veterinary Sciences, Nicolaus Copernicus University in Toruń, Lwowska 1, 87-100 Toruń, Polandapawelek@umk.pl (A.P.); milena.kulasek@umk.pl (M.K.); browsk@umk.pl (G.B.D.)

**Keywords:** BnMT, canola, endophytic bacteria, salinity, phytohormones, promoter

## Abstract

Background/Objectives: Plant metallothioneins (MTs) are low-molecular-weight proteins involved in heavy metal binding and response to stress conditions. This work aimed to analyse canola (*Brassica napus* L.) *MTs* (*BnMT1-4*) response to salinity and plant interaction with bacteria. Methods: (1) We tested germination and canola growth and development in the presence of sodium chloride and bacteria *Serratia plymuthica*; (2) We analysed phytohormones content using LC-MS/MS; (3) We identified in silico *cis*-regulatory elements in promoters of *BnMT1-4* genes; and (4) we investigated *BnMT1-4* genes’ expression in *B. napus*. Results: Under saline conditions, canola germination and plant growth were notably inhibited, whereas inoculation of seeds with *S. plymuthica* significantly stimulated the analysed physiological traits of *B. napus*. The content of auxin, abscisic acid, jasmonates, gibberellins, and salicylic acid in *B. napus* was significantly affected by salinity and modulated by *S. plymuthica* presence. The promoter regions of the *BnMT1-4* genes contain numerous regulatory elements controlled by light, hormones, and various stresses. Interestingly, the expression of *BnMT1-3* genes was down-regulated under salt stress, while *BnMT4* transcript levels increased strongly at the highest salt concentrations with and without *S. plymuthica* present. Conclusions: The results show that *BnMT* genes are differently affected by salinity and bacteria *S. plymuthica* and significantly correlate with particular phytohormones content in canola tissues, confirming the diversified functions of MTs in plant responses to changing environment.

## 1. Introduction

The Food and Agriculture Organization of the United Nations, based on data covering 85% of land area worldwide, estimates that more than 424 million hectares of topsoil (0–30 cm) and 833 million hectares of subsoil (30–100 cm) are affected by salt [1]. Although natural salinisation due to climate, lithology, and pedology occurs, it is highly advanced by anthropogenic activities, mainly irrigation, but also fertilisation and contamination of the environment with chemical pollution [2]. For important crops, including wheat (*Triticum aestivum* L.), rice (*Oryza sativa* L.), and maize (*Zea mays* L.), the yield losses due to salinity vary between 20 and 50% [3,4]. Salinity limits plant growth and development by increasing the level of reactive oxygen species, which results in oxidative damage. Moreover, salinity affects the Na^+^/K^+^ ratio and thus disturbs membrane potential, osmotic and turgor pressure, and tropisms [5]. The increased uptake of Na^+^ and Cl^−^ leads to a reduction of osmotic potential between root and soil solution and thus reduced water uptake. Also, nutrient uptake is disturbed by high concentrations of ions [6]. Plants have evolved several mechanisms to cope with high levels of salt. Salt-sensitive plants exclude most of the uptaken salt back to the soil solution. Some salt-tolerant species, including naturally adapted to saline environment halophytes and salt-tolerant nonhalophyes, adopt two strategies to survive in the saline environment: (i) salt tolerance i.e., reduction of Na^+^ uptake, excretion of Na^+^, and Na^+^ compartmentalization (e.g., accumulation of high amounts of Na^+^ and Cl^−^ in the leaves’ vacuoles), and (ii) salt avoidance through salt secretion (e.g., salt hairs), shedding of the old leaves, and succulence. Salt tolerance is also tightly coupled with reactive oxygen species (ROS) detoxification performed by various antioxidative enzymes and the accumulation of osmoprotectants such as proline, glycine, betaine, or polyphenols [7]. Halophytes are also highly tolerant to heavy metal stress. For example, *Tamarix smyrnensis* Bunge accumulated more cadmium with increasing salinity. Moreover, cadmium did not significantly affect plant growth and development. This plant excreted cadmium with salt crystals from glandular tissue [8]. Salt and heavy metal tolerance depend, to some degree, on the exact molecular mechanisms. Those mechanisms include the production of osmoprotectants, more efficient than in glycophytes antioxidant system, chelation and sequestration of ions, and possession of modified structures such as salt glands, salt bladders, and trichomes, allowing for the removal of the excess of toxic ions [9].

Metallothioneins (MTs) are low-molecular-weight, cysteine-rich, metal-binding proteins widespread among animals, plants, and fungi and are also found in some prokaryotes [10]. Plant MTs (pMTs) are diversified in terms of their primary structure, and, based on the number and arrangement of cysteines, they are divided into four types (pMT1-4) [11]. It is widely accepted that there is no single unifying function for all types of plant metallothionein. Besides the role of pMTs in zinc and copper homeostasis and detoxification of cadmium [12], pMTs are also involved in response to numerous environmental stresses such as oxidative stress [13], drought [14], cold [15], and pathogen attack [16]. In addition, a few lines of evidence support the role of pMTs in response to salinity. Increased expression in response to salt treatment and higher tolerance of transgenic *Arabidopsis thaliana* (L.) Heynh. plants in response to salt stress were also observed for rice *MT1* [17]. Heterologous expression of type 3 rice MT (*OsMT3a*) in salt-sensitive *E. coli* cells resulted in better growth of those bacteria and lower accumulation of Na^+^ compared to bacteria transformed with an empty vector. Interestingly, the expression of *OsMT3a* was induced by NaCl treatment in salinity-tolerant rice cultivars but not in salinity-sensitive ones [18]. The expression of type 2 metallothionein from halophyte *Suaeda salsa* (L.) Pall. (*SsMT2*) was induced by NaCl. Yeast cells and transgenic *A. thaliana* plants expressing *SsMT2* were more tolerant towards NaCl than wild-type organisms [19]. *A. thaliana* expressing *MT* from halophyte shrub *Halostachys capsica* (Bieb.) C. A. Mey was less sensitive to CaCl_2_ and NaCl despite higher Cd^2+^ and Na^+^ accumulation. Interestingly, transgenic plants also accumulated lower amounts of H_2_O_2_ when treated with NaCl and CdCl_2_ than wild-type plants [20].

Among other mechanisms allowing for the adaptation to salt stress are interactions with plant growth-promoting rhizobacteria (PGPR) [21]. Plant growth-promoting bacteria are an extremely important element of soil ecosystems due to their fast growth rate, high adaptability to various environments, and biochemical versatility to metabolize a wide range of natural compounds and xenobiotics [22]. PGPR can improve plant growth through various direct and indirect mechanisms, including (1) increased mineral nutrient solubilization and nitrogen fixation, (2) phytohormones production, (3) antagonism against phytopathogenic bacteria, and (4) the ability to produce 1-aminocyclopropane-1-carboxylate (ACC) deaminase enzyme, which reduces ethylene levels [23]. During salt stress, PGPR induces absorption of K^+^ and exclusion of Na^+^, forms the biofilm to reduce sodium toxicity, increases the level of antioxidants and osmoprotectants, and enhances the expression of salt stress-responsive genes [24]. For example, halotolerant bacteria *Pseudomonas* sp. ISE-12 isolated from the roots of obligatory halophyte *Salicornia europaea* L. enhanced beet (*Beta vulgaris* L.) seed germination and plant growth in the presence of NaCl [25]. Inoculation with *Serratia liquefaciens* KM4 increased *Z. mays* growth, biomass production, and nutrient uptake under salt stress [26]. Using PGPR is a cost-effective and environmentally friendly approach in agriculture, with the potential to reduce the use of chemical fertilizers and pesticides. Although multiple microbial strains have been shown to improve plant species’ growth and development in stress conditions, the number of field trials is still limited. Whether the microbes introduced to the environment will survive, thrive, and promote the growth of plants is crucially dependent on other microbes in the soil and environmental conditions [23].

*Brassica napus* L. (canola, rape, rapeseed) is a socioeconomically important oil plant belonging to the *Brassicaceae* family. Canola oil is nutritionally valuable because it contains low levels of unfavourable saturated fatty acids and substantial amounts of monounsaturated fatty acids. In addition, canola oil is a natural source of bioactive compounds such as vitamin E, phenolic acids, and phytosterols [27]. Post-extraction meal is a valuable high-protein feed for animals. Canola seeds are also an excellent raw material to produce biofuels, adhesives, surfactants, and cosmetics [28]. Global production of *B. napus* in 2020 and 2021 was almost 74 million tons and is anticipated to grow in the coming years [29]. *B. napus* is an amphidiploid (allotetraploid) that resulted from the interspecific hybridization between cabbage vegetable (*Brassica oleracea* L.) and turnip (*Brassica rapa* L.). Polyploidization generates great genetic and phenotypic variance and thus is one of the major forces of plant evolution. However, establishing the link between phenotype and genotype is not trivial [30]. This study aimed to analyse the potential role of *B. napus* metallothioneins (BnMT) in response to salt stress. Despite over 30 years of research, since the discovery of pMTs in wheat germs in 1987 [31], the physiological roles of these proteins in plant stress response remain elusive. In *B. napus*, four types of metallothioneins (BnMT 1-4) were identified and were differentially expressed during germination and in the organs [32]. Encouraged by the previous results showing the possibility of enhancing canola growth and development in stress conditions by applying plant growth-promoting microorganisms, we tested the potential of *Serratia plymuthica* for enhancing *B. napus* growth and development in salt stress. To gain deeper insight into the molecular mechanism of interaction between plants and PGPR in salt stress, we analysed the expression of *BnMT1-4* and the content of selected phytohormones in canola plants.

## 2. Materials and Methods

### 2.1. Plant Material and Preparation of Bacterial Inoculum

The bacterial strains *Serratia plymuthica* (GeneBank NCBI acc. No FJ786078), *Serratia liquefaciens* [33], and *Massilla timonae* [34] were obtained from the collection of Prof. Katarzyna Hrynkiewicz from the Department of Microbiology at the Nicolaus Copernicus University in Toruń. To obtain bacterial inoculum, bacteria were grown on agar R2A (Difco) medium (18 g /L) at 24 °C and then in liquid R2A medium at 24 °C with shaking at 180 rpm/min. The density of bacterial culture was checked spectrophotometrically at 600 nm (OD_600nm_) (SmartSpec Plus, BioRad, Hercules, CA, USA) and adjusted to a value of 5 × 10^7^cfu/mL. The prepared bacterial suspension inoculated *B. napus* seeds of winter cultivar Harry (Obrol, Kruszewnia, Poland).

### 2.2. Bacteria Growth in the Presence of NaCl

Subsequently, 50 mL of R2A liquid medium was inoculated with *S. plymuthica*, *S. liquefaciens*, and *M. timonae* and incubated for 20 h at 24 °C. Overnight cultures of bacteria were diluted (1:100, *v*:*v*) in R2A medium to an optical density (OD_600nm_) of ca. 0.2. Sodium chloride was added to a final concentration of 50, 100, 150, 200, 250, 300, 350, 400, 450, 500, 550, and 600 mM. Cultures without NaCl served as controls. Bacteria cells were collected every hour for 7 hours, and then after 12, 24, and 48 h from the start of the experiment, OD_600nm_ was measured (SmartSpec Plus, BioRad, Hercules, CA, USA). The growth rate of bacteria cultures was expressed as the slope of the linear proportion of the growth curve, calculated using Microsoft Excel. The experiment was performed three times.

### 2.3. B. napus Seed Germination, Seedling and Plant Growth in the Presence of NaCl and S. plymuthica

*B. napus* seeds were surface sterilised in a mixture of 30% hydrogen peroxide and 96% ethanol (1:1, *v*:*v*) for 5 min and rinsed 10 times with sterile water. The seeds were inoculated with the suspension of bacteria at a density of 5 × 10^7^ cfu/mL for 30 min and placed in Petri dishes (90 mm diameter) on filter paper moistened with 5 mL of sterile water (control) or 5 mL of 50 mM, 100 mM, 150 mM, and 200 mM NaCl. Seeds non-inoculated with bacteria served as control. Seeds were incubated in a 16 h darkness/8 h light photoperiod at 25 °C for 6 days. At the 17th, 20th, and 48th hour of germination, the number of germinated seeds (i.e., seeds with visible radicle) was counted. The fresh and dry biomass, the length of hypocotyls, and roots of 6-day-old seedlings were measured. For *BnMT1-BnMT4* genes expression analysis and analysis of phytohormones content, 6-day-old shoots were frozen in liquid nitrogen and stored at −80 °C until further analyses were performed. The experiment was performed in triplicate.

Bacteria-inoculated seeds were also grown in a mixture of soil, sand, and vermiculite (2:1:1, *w*:*w*:*w*) pre-soaked with water (control) or 150 mM, 300 mM, and 450 mM NaCl before seeds sowing. Each plant was grown separately in a 2-L pot. Plants were watered twice a week with ~250 mL of tap water and with 200 mL of ½ Hoagland solution every two weeks. After 14 weeks, the length of shoots, the number of internodes, and the fresh and dry mass of shoots were examined. The experiment was repeated three times with five plants per treatment.

### 2.4. Phytohormones Content

To determine the concentrations of endogenous indole-3-acetic acid (IAA), abscisic acid (ABA), jasmonic acid (JA), methyl jasmonate (JAMe), gibberellins (GA_3_, GA_7_), and salicylic acid (SA), mass spectrometry integrated with liquid chromatography (LC-MS/MS) and the QuEChERS-based extraction method [35] were used. Plant material was ground in liquid nitrogen, and samples (100 mg) were shacked overnight at 8 °C in an extraction buffer: 80% acetonitrile, 5% formic acid (FA), 15% water, 1 mM butylhydroxytoluene (BHT), and stable isotope-labelled internal standards (5 ng/mL d2IAA; 5 ng/mL d6ABA; 10 ng/mL d5JA; 10 ng/mL d2JAMe; 10 ng/mL d2GA3; 10 ng/mL d2GA7; 10 ng/mL d4SA). The further isolation procedure was performed as described previously [36].

The quantifications of phytohormones were determined using the LC-MS/MS Nexera UHPLC and LCMS-8045 integrated system (Shimadzu Corporation, Kyoto, Japan). The samples were separated chromatographically on a reversed-phase C18 column (150 × 2.1 mm, 2.6 µm, Kinetex^®^). The mobile phase water with 0.1% FA (*v*:*v*) (A) and methanol with 0.1% FA (*v*:*v*) (B) were used. The separation was carried out on a linear gradient of 40–90% (*v*:*v*) methanol for 7 min at 30 °C. The flow rate was 0.4 mL/min, and the injection volume was 5 µL. In mass spectrometry, the samples were subjected to negative and positive electrospray ionization (ESI) (4 kV voltage). For phytohormone analysis, multiple reactions monitoring (MRM) with the LabSolutions workstation for LCMS-8045 was used. Three biological repetitions were conducted, and the data are presented as mean ± standard deviation (SD).

### 2.5. Identification of Cis-Regulatory Elements in Promoters of BnMT Genes

Genomic sequences of *BnMT* genes were retrieved from the NCBI nucleotide sequences database (www.ncbi.nlm.nih.gov/nuccore, accessed on 20 January 2024). The promoter sequences of 1500 bp length were analysed. For some genes, i.e., *BnMT1*_C8, *BnMT2*_A1, *BnMT2*_C1, *BnMT2*_A3a, and *BnMT4*_A3, shorter regions of genomic DNA were analysed due to the presence of the coding region of another gene. The *cis*-regulatory sequences were identified using the PlantCARE database (http://bioinformatics.psb.ugent.be/webtools/plantcare/html/, accessed on 14 February 2024).

### 2.6. BnMT1-BnMT4 Genes Expression Analysis by qRT-PCR

According to the manufacturer’s protocol, total RNA from *B. napus* seedlings was isolated using RNeasy Plant Mini Kit (Qiagen, Hilden, Germany). The quality and quantity of RNA were analysed via spectroscopic measurement (NanoDrop Lite, Thermo Fisher Scientific, Waltham, MA, USA) and agarose gel electrophoresis. One µg of total RNA was treated with one U of DNase I (Thermo Fisher Scientific, Waltham, MA, USA). Then, RNA free of any genomic DNA contamination was used for the reverse transcription reaction using the NG dART RT kit (EURx, Gdańsk, Poland) with 200 ng of random hexamers and 300 ng of oligo(dT)_20_ according to the manufacturer’s protocol. An RT reaction without reverse transcriptase was performed to verify the lack of contamination with genomic DNA for each sample. Based on available literature data [37], SAND, RPL, and PP2A were chosen as potential reference genes. The genes were analysed using the RefFinder tool [38], and, based on the analysis, PP2A (XM_013790313.3) was selected. All primers are listed in Table 1. Real-time PCR was performed using a LightCycler 480 II Instrument (Roche, Mannheim, Germany). The reaction mixture consists of 4 μL of 20× diluted cDNA, 1× LightCycler 480 SYBR Green I Master, and 0.5 μM forward and reverse primers each. The following PCR conditions were used: 95 °C for 5 min for initial denaturation followed by 40 cycles of 95 °C for 10 s, 59 °C for 20 s, and 72 °C for 20 s. Melt curve analysis was used to verify the specificity of the PCR reaction. The relative gene expression was calculated using LightCycler 480 software version 1.5.1.62 (Roche, Mannheim, Germany). The reaction was performed in three technical replicates for each of the three biological replicates.

### 2.7. Statistical Analysis

Data analysis was conducted using one-way ANOVA followed by Tukey’s post hoc multiple comparisons test. Visualisation was performed in R using the ‘ggplot2’, ‘multcomp’, and ‘multcompView’ packages. Results are presented as mean values from three replicates accompanied by standard deviation (SD). Significant differences between treatments were identified at *p* ≤ 0.05. Correlation analysis was performed and visualised using the ‘Hmisc’ and ‘corrplot’ packages in R.

## 3. Results

### 3.1. Tolerance of S. plymuthica, S. liquefaciens, and M. timonae to Sodium Chloride

**Figure 1:**
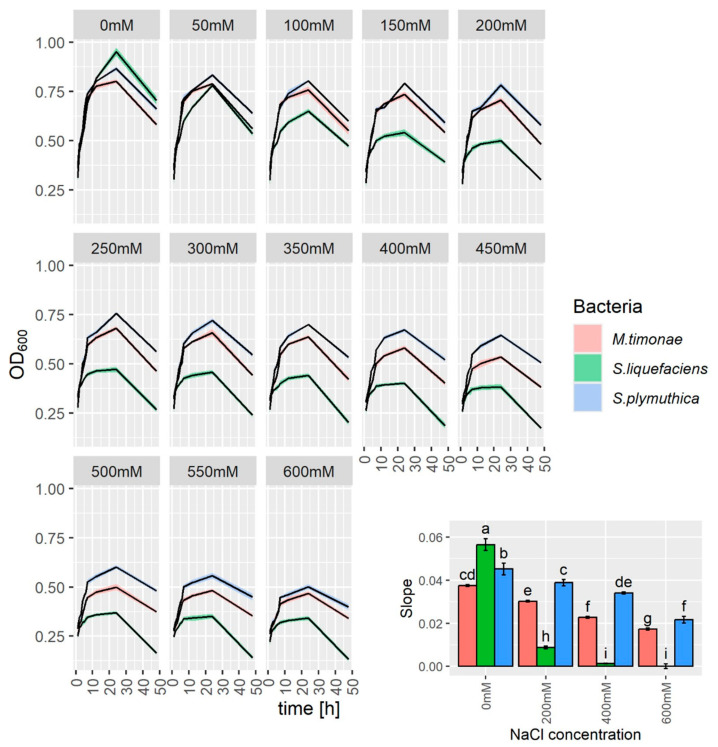
The growth of *S. plymuthica* (blue line), *S. liquefaciens* (green line), and *M. timonae* (red line) grown in R2A liquid medium containing NaCl (50–600 mM) or without NaCl (0 mM) shown as OD_600nm_. Solid lines represent means, and ribbons represent SD. The relative growth rate (bar plot in the lower right corner) is expressed as the slopes of the bacterial growth curves obtained from plotting optical density against time. Data are means of three independent experiments ± SD. Different letters indicate statistically significant differences (*p* < 0.05, one-way ANOVA followed by Tukey’s post hoc test).

To verify the level of the tolerance of bacteria *S. plymuthica*, *S. liquefaciens*, and *M. timonae* to the presence of sodium chloride, the growth of bacteria in liquid culture supplemented with 0–600 mM NaCl was monitored (Figure 1). The most sensitive to NaCl-treatment was *S. liquefaciens* i.e., the growth rate of this bacteria was severely inhibited by 200 mM NaCl. The growth of the other two bacteria was not completely inhibited even when the final concentration of NaCl in the medium was 600 mM i.e., the growth of *S. plymuthica* was 2.1 times slower, and the growth of *M. timonae* was 2.2 times slower than in control (0 mM NaCl) (Figure 1). Since the *S. plymuthica* was the least sensitive to NaCl and this strain has already been shown to promote *B. napus* growth (unpublished data), it was selected for further study.

### 3.2. B. napus Seed Germination and Seedling Growth in the Presence of Sodium Chloride and S. plymuthica

**Figure 2:**
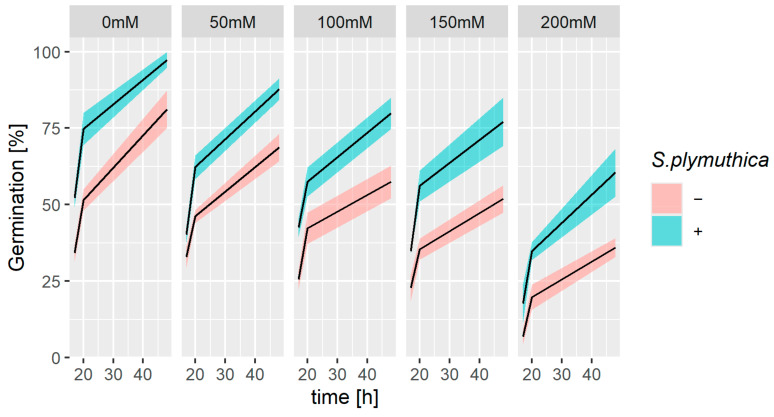
Effect of sodium chloride and/or the presence of *S. plymuthica* on the germination rate of *B. napus*. Solid lines represent means and ribbons represent SD from three independent replicates for 60 seeds.

*B. napus* seed germination (Figure 2) was significantly affected by salinity. On the other hand, the presence of *S. plymuthica* significantly improved the germination and growth of both NaCl-treated and control seeds and seedlings. The germination ratio of seeds incubated in 200 mM NaCl was over 2-fold lower than the germination ratio of control seeds (Figure 2). *S. plymuthica* significantly stimulated seed germination; i.e., in 200 mM NaCl, only 35% of seeds germinated, whereas inoculation with bacteria increased this value to 60%. In control conditions, the germination ratio increased from 80% without bacteria inoculation to almost 98% in the presence of *S. plymuthica* (Figure 2).

**Figure 3:**
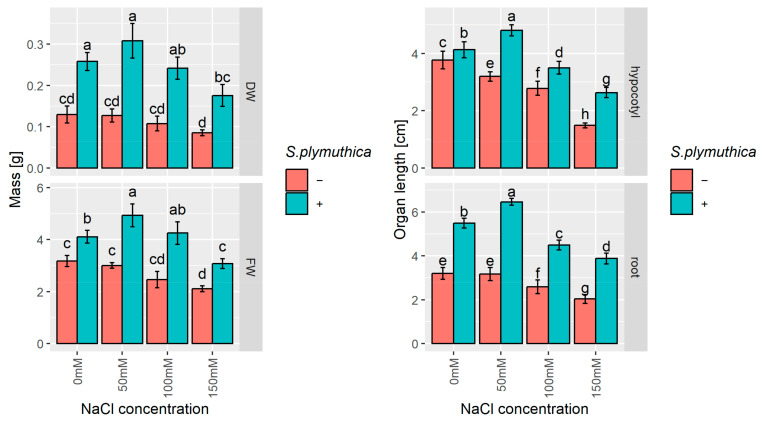
The effect of sodium chloride and/or *S. plymuthica* on the growth (fresh and dry biomass, length of hypocotyls and roots) of *B. napus* 6-day-old seedlings. Bars represent means ± SD from three independent replicates. Different letters indicate statistically significant differences (*p* < 0.05, one-way ANOVA followed by Tukey’s post hoc test).

Sodium chloride also negatively affected further stages *of B. napus* seedling growth (Figure 3). Some seeds germinated in the presence of 200 mM NaCl; however, the further growth of seedlings was severely inhibited. Therefore, they were excluded from further analyses. Significant root length reduction was observed in seedlings grown in 100 and 150 mM NaCl, i.e., 1.2-fold and 1.6-fold shorter than in control, respectively. In contrast, the hypocotyl growth was notably inhibited even by 50 mM NaCl, i.e., 1.2-fold shorter than in control (Figure 3). *S. plymuthica* significantly improved the growth of *B. napus* seedlings in NaCl and non-saline conditions, and the effect was most noticeable in the roots of seedlings grown from seeds inoculated with bacteria, which were 1.7–2 times longer than those of seedlings from non-inoculated seeds. For hypocotyls, growth stimulation by *S. plymuthica* was more evident with higher salt concentrations. Evidently, the roots and hypocotyls of seedlings grown from bacteria-inoculated seeds in the presence of 50 mM NaCl were approx. 1.2 times longer than the organs of seedlings grown from bacteria-inoculated seeds in non-saline water (Figure 3). Treatment of canola with NaCl and with *S. plymuthica* also affected seedlings’ fresh and dry biomass. At 150 mM NaCl, fresh and dry biomass was approx. 1.5-fold lower compared to the weights of control seedlings. The biggest stimulation of fresh and dry biomass production by seedlings growing in the presence of *S. plymuthica* was observed at 50 mM NaCl concentration. A 1.6-fold and 2.4-fold increment in fresh and dry biomass was observed in seedlings growing in the presence of bacteria and 50 mM NaCl compared to the biomass of non-inoculated seedlings growing in 50 mM NaCl.

### 3.3. The Growth of B. napus Plants in the Presence of Sodium Chloride and S. plymuthica

**Figure 4:**
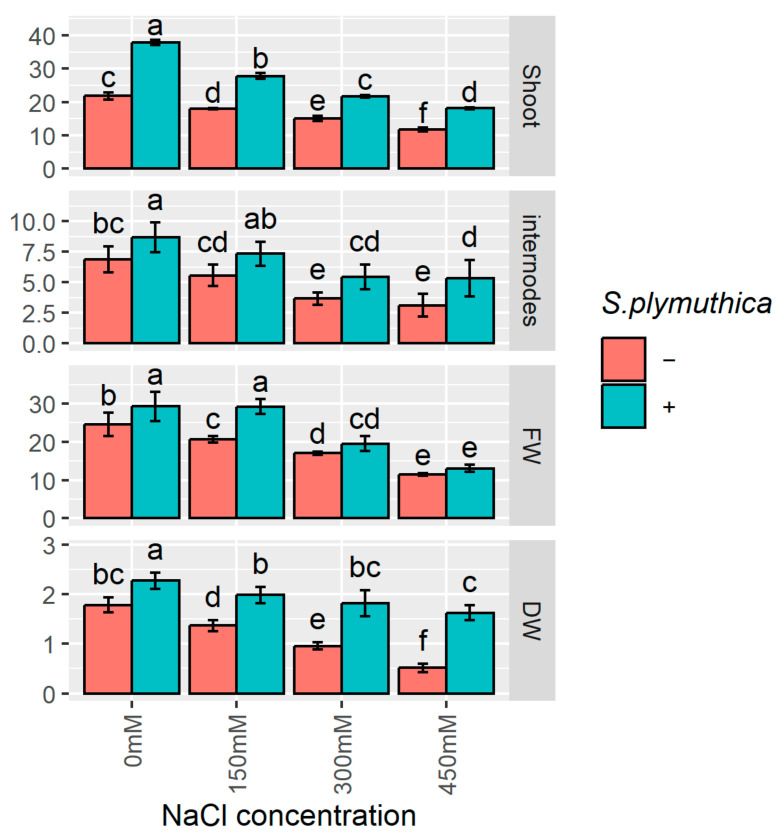
The effect of sodium chloride and/or *S. plymuthica* on the growth (fresh and dry biomass, length of shoot, and the number of internodes) of *B. napus* 14-week-old plants. Bars represent means ± SD from three independent replicates. Different letters indicate statistically significant differences (*p* < 0.05, one-way ANOVA followed by Tukey’s post hoc test).

To examine the effect of salinity and *S. plymuthica* on further stages of *B. napus* plant growth and development, plants were grown in soil for 14 weeks. Shoot length, the number of internodes, and fresh and dry mass of shoots were then analysed (Figure 4). It is apparent that salt negatively affects the growth of *B. napus* plants. At the highest NaCl concentration of 450 mM, the hypocotyl length is 1.9-fold lower, the fresh biomass is 2.1-fold smaller, and the dry biomass is 3.5-fold smaller than those of control plants. The mean number of internodes decreased from almost 7 in control plants to 3 in 450 mM NaCl-treated plants. Moreover, the great positive impact of *S. plymuthica* on *B. napus* growth and development is visible (Figure 4). Although salt negatively affected the growth of plants even in the presence of bacteria, a significant increase in all tested parameters was observed compared to plants growing in the same salt concentration without bacteria. For example, in 450 mM NaCl, the inoculation with *S. plymuthica* increased the shoot length 1.5-fold, the number of internodes 1.7-fold, fresh biomass 1.3-fold, and dry biomass 3.2-fold.

### 3.4. The Content of Phytohormones

To check the hormonal regulation under the influence of salt stress and *S. plymuthica* inoculation, the levels of selected phytohormones in *B. napus* seedlings were analysed.

**Figure 5:**
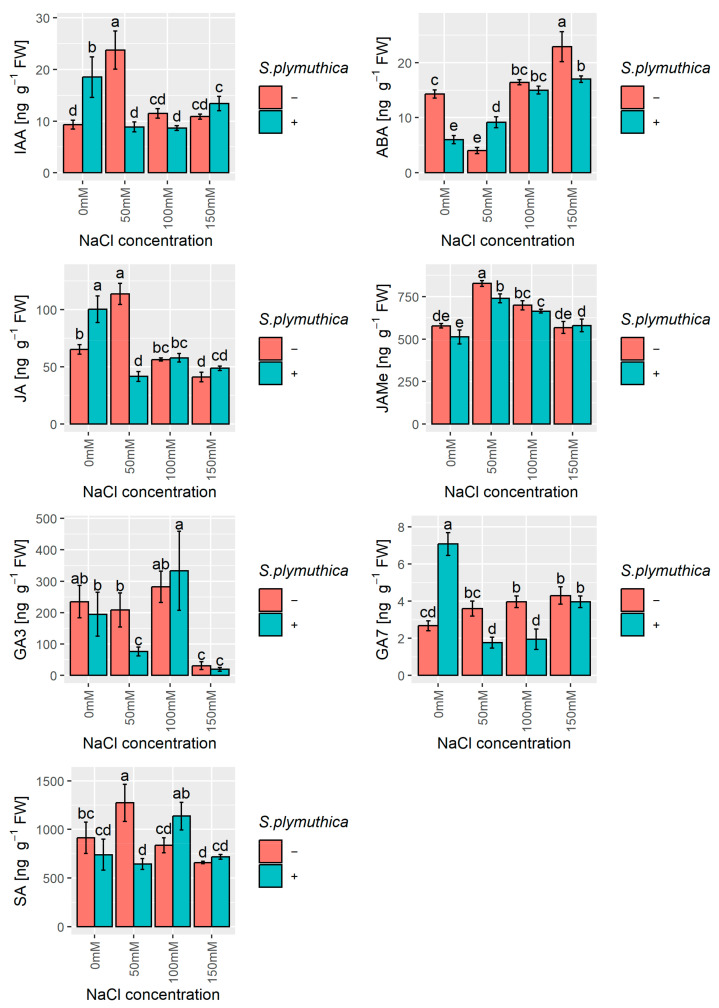
The effect of sodium chloride and/or *S. plymuthica* on the phytohormone (IAA, ABA, GA3, GA7, JA, JAMe, SA) concentrations in *B. napus* 6-day-old shoots. Bars represent means ± SD from three independent replicates. Different letters indicate statistically significant differences (*p* < 0.05, one-way ANOVA followed by Tukey’s post hoc test).

Salinity significantly influenced IAA level only at 50 mM NaCl concentration, causing a 2.5-fold increase compared to the control without salt (Figure 5). In the presence of *S. plymuthica*, IAA content increased 2-fold in no-salt conditions and decreased 2.7-fold in 50 mM NaCl concentration.

Two specific salt concentrations significantly modulated ABA content in *B. napus* seedlings. The level of ABA decreased 3.5-fold in 50 mM NaCl and increased 1.6-fold in 150 mM NaCl compared to the controls without salt. *S. plymuthica* inoculation decreased the ABA content 2.4-fold in the absence of NaCl and 1.3-fold in the presence of 150 mM NaCl compared to controls in the same respective salt concentrations without bacteria. However, *S. plymuthica* inoculation in 50 mM NaCl caused a significant rise by 2.3-fold in the ABA level compared to controls in 50 mM NaCl without bacteria (Figure 5).

JA and its derivative methyl ester (JAMe) are collectively called jasmonates (JAs). JAs regulate plant growth and development, including embryonic axis elongation and root and flower formation, as well as improving plant tolerance to different abiotic stresses [39]. In *B. napus* seedlings, the JA level was affected by salinity depending on NaCl concentration. Compared to the control without salt, in the presence of 50 mM NaCl, JA level increased by 1.7-fold, while in higher NaCl concentrations (100–150 mM), it decreased by 1.6-fold (Figure 5). In the presence of *S. plymuthica*, JA content increased 1.5-fold in no-salt conditions and decreased 2.7-fold in 50 mM NaCl. The influence of salinity and *S. plymuthica* inoculation on JAMe content was similar to the observed JA changes. The exception was noted for no-salt conditions, where *S. plymuthica* caused a slight decrease in JAMe level compared to the control without bacteria.

We analysed two bioactive gibberellins in the plant materials: gibberellic acid (gibberellin A3, GA_3_) and gibberellin A7 (GA_7_). The salinity of 150 mM NaCl strongly decreased the GA_3_ level 7.6-fold compared to the control without salt (Figure 5). *S. plymuthica* significantly changed the GA_3_ level only in 50 mM NaCl, causing a 2.7-fold drop compared to the control in the same NaCl concentration without bacteria. On the other hand, GA_7_ content in *B. napus* seedlings was increased significantly by salinity in all studied NaCl concentrations (50–150 mM) compared to the control without salt (Figure 5). The presence of *S. plymuthica* in no-salt conditions greatly increased, by 2.6-fold, the GA_7_ level compared to the no-salt control without bacteria. However, *S. plymuthica* inoculation at 50 mM NaCl and 100 mM NaCl decreased GA_7_ content 2-fold compared to their controls without bacteria.

SA is crucial in plant defence against pathogens and is involved in plant-microbe symbiotic interactions [40]. In our study, the lowest (50 mM) and highest (150 mM) NaCl concentrations by 1–4-fold increased and decreased, respectively, the SA level compared to their controls without salt (Figure 5). However, in the presence of *S. plymuthica*, SA content in *B. napus* seedlings decreased 1.9-fold in 50 mM NaCl and increased 1.3-fold in 100 mM NaCl compared to their controls without bacteria.

### 3.5. In Silico Analysis of Promoter Sequences of BnMT Genes

**Figure 6:**
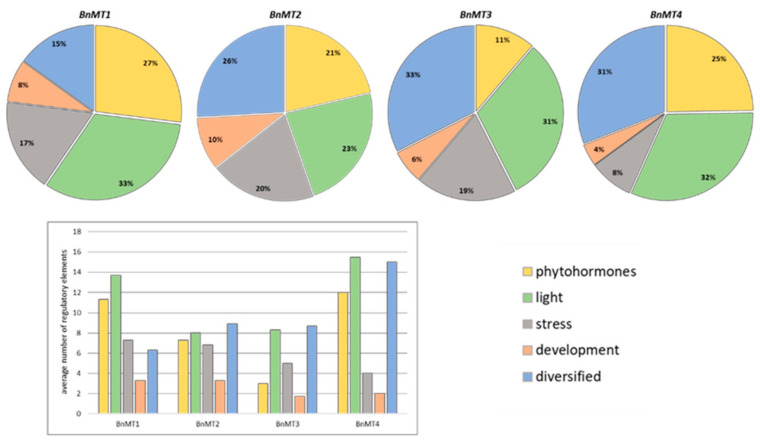
The pie charts show frequencies of *cis*-acting elements in *BnMT1-4* gene promoters grouped according to their predicted functions. The bar chart shows the average number of regulatory elements in the promoters of *BnMT1-4* genes (the total number of regulatory elements of each type divided by the total number of genes). The detailed lists of motifs and the number for each promoter are presented in Supplementary Table S1.

To gain insight into the putative functions of BnMT1-4, in silico analysis of promoter sequences using the PlantCARE database was performed (Figure 6 and Table S1). In total, 61 putative *cis*-regulatory elements (CREs) were identified (Table S1), which were further divided into five types based on the predicted functions (Figure 6). This analysis revealed the presence, in the promoter sequences, of *BnMT1-4* genes of multiple CREs involved in phytohormone signalling, light response, biotic and abiotic stress responses, and development. The category diversified includes some elements with unknown or multiple functions. Still, the most abundant in this category were MYB (response to drought and ABA) and MYC (response to drought, cold, and ABA) elements. The light-responsive elements were the most abundant in the promoters of *BnMT1* and *BnMT4* genes. For the promoters of *BnMT2* and *BnMT3*, gene CREs belonging to the category diversified were the most frequent (Figure 6, upper panel). The ABA-responsive element ABRE was the most common element, not found in only two promoters (*BnMT2*_C2 and *BnMT3*_C5). In the promoter of *BnMT1*_A10, 13 ABRE elements were identified; this was the highest number of one type of element found in one promoter (Table S1). Promoters of *BnMT1* and *BnMT4* genes were richer in CREs than promoters of *BnMT2* and *BnMT3*. The highest average number of CREs involved in the response to phytohormones and light, as well as diversified CREs, were observed in promoters of *BnMT4* genes. The average number of stress-related regulatory elements was the lowest in promoters of *BnMT4* and the highest in the promoters of *BnMT1* (Figure 6, lower panel).

### 3.6. Analysis of BnMT1-4 Gene Expression in B. napus Seedlings in Response to Sodium Chloride and S. plymuthica

**Figure 7:**
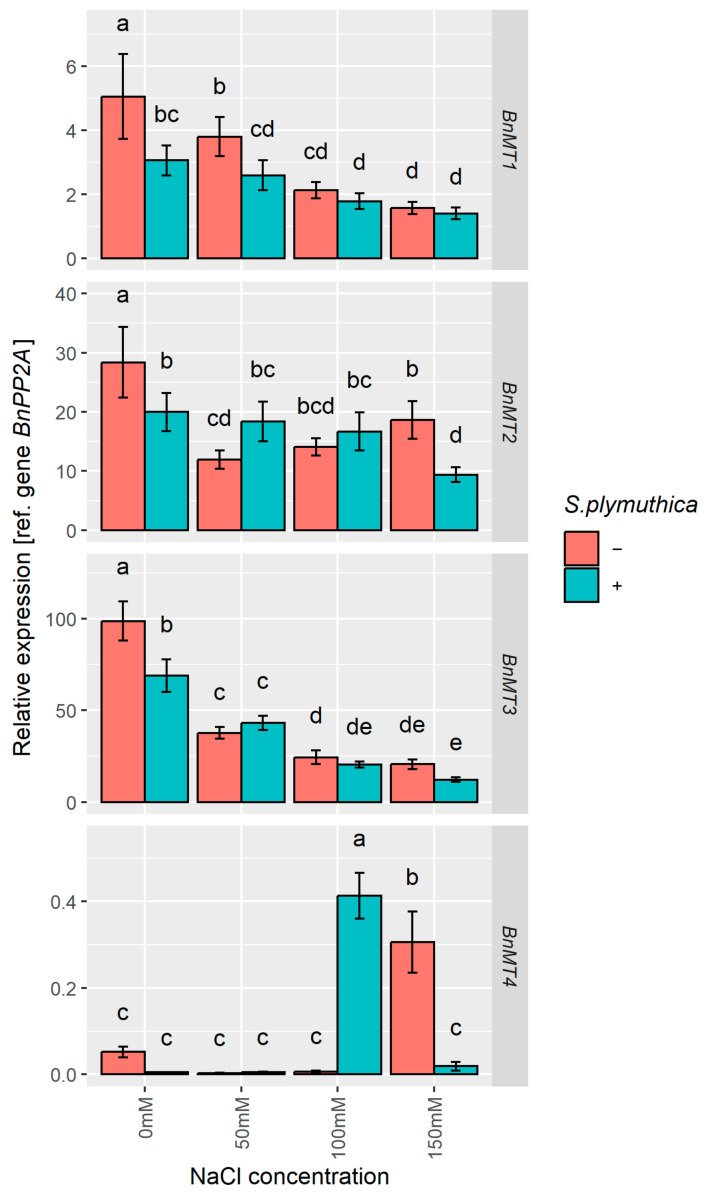
The effect of sodium chloride and/or *S. plymuthica* on the relative expression of *BnMT1-4* genes in *B. napus* 6-day-old shoots. Bars represent means ± SD from three independent replicates. Different letters indicate statistically significant differences (*p* < 0.05, one-way ANOVA followed by Tukey’s post hoc test).

The expression of all tested *BnMT* genes was affected by salt and *S. plymuthica* (Figure 7). *S. plymuthica* decreased the expression of *BnMT1-4* in seedlings grown without NaCl. The expression level was lower by 1.9-fold for *BnMT1*, 1.4-fold for *BnMT2*, 2.3-fold for *BnMT3*, and 10-fold for *BnMT4*. Moreover, the expression level of *BnMT1-3* was decreased by NaCl in a dose-dependent manner, with the exception of *BnMT2*, for which the expression level in seedlings treated with 150 mM NaCl was higher than in seedlings treated with 50 mM NaCl. The inoculation of seeds with *S. plymuthica* generally did not significantly affect the expression level of *BnMT1-3* in seedlings grown in the presence of NaCl. Only the mRNA of *BnMT1* was lower after inoculation with bacteria in seedlings grown in 50 mM NaCl, and the same was observed for *BnMT2* in seedlings grown in 150 mM NaCl. NaCl up-regulated the expression level of *BnMT4*, but only at the highest concentration, i.e., the expression in seedlings treated with 150 mM NaCl was 6.2-fold higher than in control seedlings. Moreover, the expression of *BnMT4* in seedlings that grew from *S. plymuthica* inoculated seeds grown in the presence of 100 mM NaCl was 100 times higher than in seedlings that grew from bacteria-inoculated seeds grown in control (0 mM NaCl) conditions. Interestingly, the mRNA level of *BnMT4* in seedlings that grew from uninoculated seeds grown in the presence of 100 mM NaCl and in seedlings that grew from inoculated seeds grown in the presence of 150 mM NaCl was lower than in the respective control seedlings (Figure 7).

### 3.7. Correlations Between Growth Parameters, Phytohormone Content and BnMTs Gene Expression

**Figure 8:**
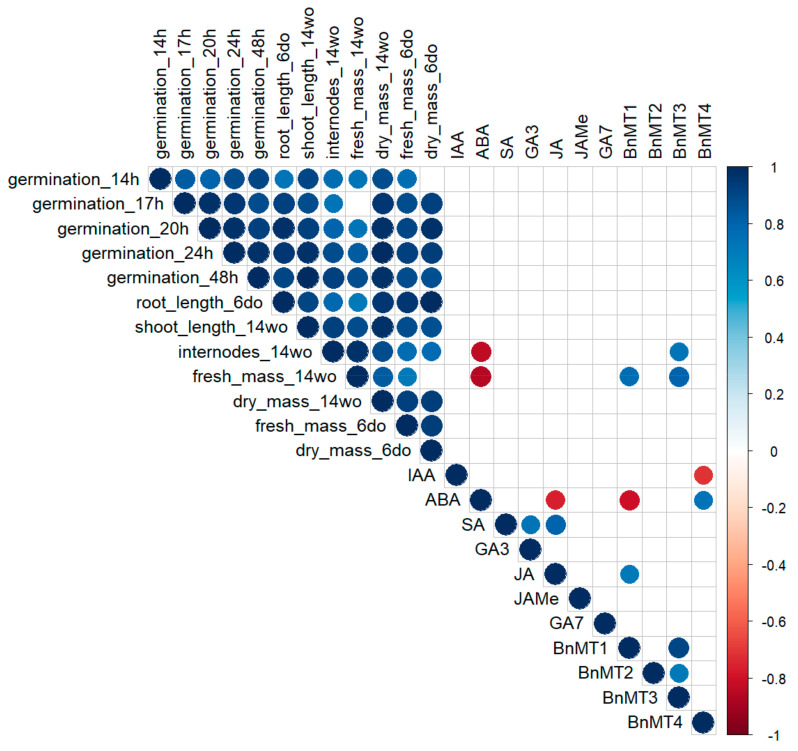
Spearman correlations between *BnMT1-4* gene expression levels, phytohormone content, and plant traits measured during germination, seedling growth (6 days old), and mature plant development (14 weeks old). Only significant relationships are shown (*p* < 0.05).

Correlation analysis shows a positive correlation between germination and the studied growth parameters for *B. napus* seedlings (root length, fresh and dry biomass) and mature plants (internodes number, shoot length, fresh and dry biomass). Moreover, most of the studied growth parameters of 6-day-old seedlings correlate positively with the growth parameters of 14-week-old plants (Figure 8). Among the phytohormones tested, the ABA level correlates negatively with the JA content in canola seedlings, whereas the SA level correlates positively with the GA_3_ and JA levels. Additionally, ABA negatively correlates with the number of internodes and the fresh weight of 14-week-old canola plants. The expression of selected *MT* genes in canola seedlings positively correlates with each other, i.e., *BnMT1* correlates with *BnMT3*, which correlates with *BnMT2*. Expression levels of *BnMT1* and *BnMT3* correlated positively with internode number and fresh biomass of 14-week-old canola plants. Moreover, in *B. napus* seedlings, the expression of *BnMT1* was negatively correlated with ABA level, whereas *BnMT4* expression correlated positively with this hormone. However, in canola seedlings, the expression level of *BnMT1* correlated positively with JA content. The only significant correlation (negative) with IAA was observed for *BnMT4* expression in 6-day-old rapeseed seedlings.

## 4. Discussion

Salinity stress, particularly at high levels, is widely known to negatively affect seed germination, seedling establishment, growth and reproduction capacity, and yield of several plant species [41,42,43,44,45,46]. However, *B. napus* is reported to tolerate moderate salinity levels [47]. It has been identified that low concentration of 25 mM NaCl treatment enhance canola seedling growth to some extent, whereas high concentrations of 50 and 100 mM NaCl treatments adversely affect seedling growth [48]. Rhizosphere bacteria such as *S. plymuthica*, *S. liquefaciens*, and *M. timonae* can potentially improve plant productivity under different stress conditions [49,50,51,52]. Strains of *Serratia* spp. are reported to have stimulated the growth of plants such as pepper (*Capsicum annuum* L.), eggplant (*Solanum melongena* L.), cucumber (*Cucumis sativus* L.), and petunia (*Petunia* × *hybrida*) in salt and water stress conditions [53,54,55,56]. The beneficial effects of *Seratia* inoculation are related to the activation of various processes in plant cells, such as phytohormone production, Zn solubilization, modulation in the plant antioxidant system, and gene expression [57]. Dąbrowska et al. [58] showed that in normal and salt-stress conditions, PGPR, especially *S. plymuthica*, promoted canola growth and alleviated stress levels by inducing a stringent response. A similar observation was made for canola and halotolerant bacteria *Pseudomonas stutzeri* ISE 12 [59].

Phytohormones are crucial regulators of plant survival under various stress conditions [60]. Our study, assessing the content of the main phytohormones in canola seedlings growing in salt stress conditions with and without *S. plymuthica* inoculation, showed that these biotic and abiotic factors significantly modulate plant hormonal balance. Similar changes in IAA, JA, and SA levels were generally observed in our study and may suggest crosstalk between these phytohormones in the studied conditions. ABA, on the other hand, showed a different pattern of changes in the studied conditions. Furthermore, statistical analysis showed a significant positive correlation between the contents of SA and JA and a negative correlation between ABA and JA. Similar to our observations in this study, several reports demonstrate specific changes in the phytohormone levels under salinity conditions and after bacterial inoculation. ABA levels increased in tobacco (*Nicotiana tabacum* L.) plants under salt stress, whereas the IAA content was only lightly affected [61]. In 8-week-old shoots of *Centaurium erythraea* Rafn under graded NaCl concentrations, IAA levels decreased in all NaCl concentrations. However, with increasing NaCl concentrations, ABA and JA contents increased while SA content decreased [62]. Reduced SA level under salt stress was also observed in rice seedlings and tomato plants [63,64]. Exogenous SA application may help plants cope with toxic ion accumulation under salt stress by modulating cellular antioxidant defence, nitrogen metabolism, accumulation of organic osmoregulators such as proline, and photosynthesis [65]. SA application is reported to enhance the colonization rate of the endophyte *Penicillium resedanum* LK6 in red pepper roots, whereas *S. plymuthica* inoculation promotes watermelon seedling growth and enhances resistance to *Fusarium* wilt by activating the JA and SA synthesis pathways [66,67]. Similarly, enhanced growth of potato plants inoculated with rhizobacteria *Azospirillum brasilense* or *Ochrobactrum cytisi* is associated with increased auxin concentrations and decreased ABA content in the plant tissue [68]. In the rhizosphere, microbes utilize various nutrients released by plant roots and, in turn, synthesize biologically active compounds, including phytohormones such as auxins, gibberellins, ABA, JA, cytokinins (zeatin), and SA [69]. It is reported that approximately 80% of rhizobacteria synthesize IAA; thus, plant growth stimulation by rhizobacteria may occur largely by IAA-related processes [70]. Rhizobacteria can also catabolise specific phytohormones such as SA and IAA [71]. This indicates that rhizobacteria can regulate the hormonal balance of plants and, thus, could improve their stress tolerance. It is well-documented that particular phytohormones cross-talk with others and alter their biosynthesis during response to salinity stress, determining the final mechanism of plant stress tolerance [72].

It is widely accepted that pMTs fulfil other functions beyond micronutrient (e.g., Zn) homeostasis and toxic metal (e.g., Cd) detoxication, such as the response to abiotic and biotic stresses [14,15,17,20]. The question of whether the role of pMTs is specific and limited to selected types of stresses or whether pMTs are part of a general stress response is still open. The role of pMTs as general stress response proteins is supported by the fact that MTs act as antioxidants because metallated and reduced thiol (-SH) groups of cysteines can be oxidised by reactive oxygen species. Moreover, MTs, by binding Cu^+^, limit the Fenton reaction, lowering ROS production [73]. On the other hand, the occurrence of multiple specific stress response elements in *BnMT* promoters analysed in this study supports the specific roles of pMTs in stress tolerance. Similarly, studies of the promoter sequences of the metallothionein genes of *A. thaliana* and rice showed that they contain multiple *cis*-elements involved in the response to several abiotic factors, including environmental stresses and phytohormones [74]. In our study, in silico analysis of canola *BnMTs* promoters showed the potential role of these genes in response to salinity/osmotic stresses regulated by phytohormones, especially ABA. Our results showed that among the most frequently occurring CREs in the *BnMT1-4* promoters were elements such as MYB, ABRE (ABA-responsive element), G-box (light-responsive *cis*-element), and MYC. These CREs, classified into various functional groups, are largely connected to ABA response and biotic interactions [75]. The AREs (anaerobic responsive elements) essential for anaerobic induction were other frequently occurring CREs in the *BnMT1-4* promoters. This indicates the possible involvement of BnMTs in cellular redox homeostasis [76]. Similarly, one of the first in silico and functional analyses of rice *OsMT2b* promoter showed the presence and inducibility of salt-responsive elements in the promoter region of this gene [77]. The ABRE elements are also widely spread in 10 *MeMT* genes from cassava (*Manihot esculenta* Crantz.) [78]. Numerous *cis*-acting elements supporting the responses of *MT* genes to various stresses are also identified in *Brassica rapa* [79], rice [80], oat (*Avena sativa* L.) [81], and cotton (*Gossypium hirsutum* L.) [76].

The in silico promoter analysis of *BnMTs* was followed by examining their mRNA levels in canola seedlings under salt stress and after *S. plymuthica* inoculation. We showed that in the absence of NaCl, inoculation with *S. plymuthica* decreases the expression of *BnMT1-4* genes. Moreover, NaCl decreased the expression level of *BnMT1-3* in a dose-dependent manner, but the *BnMT4* mRNA level was up-regulated in the highest (150 mM) NaCl concentrations. *S. plymuthica* inoculation caused an additional increase in *BnMT4* transcript amount at 100 mM NaCl. Existing reports show that the expression of *MTs* in plants can be differently affected by bacteria and/or salt stress. Inoculation of basket willow (*Salix viminalis* L.) with *Bacillus cereus* elevated relative *MT1* gene expression level in leaves [82]. Increased expression of *MT1* from *Casuarina glauca* Sieber ex Spreng. was also reported after its infection with the pathogenic bacteria *Xanthomonas campestris* [83]. Similar to our study results, *A. thaliana MT1c*, *MT2a*, *MT2b*, and *MT3* were reported to be down-regulated by salt stress, while type 4 *MTs*, *MT4a*, and *MT4b* were up-regulated [84]. Based on available experimental data, it can be concluded that different types of pMTs fulfil diversified roles, but the picture is still unclear. The differential regulation of pMTs expression in response to stress was shown in various plant species, including oat [85], rice, *A. thaliana* [84], and canola [86]. Moreover, the role of a particular pMT could be tissue specific. For example, under drought stress, the expression of oat *AsMT3* was down-regulated in the shoots but up-regulated in the roots [14]. For a long time, the expression of type 4 pMTs was believed to be restricted to developing and mature seeds and then declines rapidly after the start of germination. However, numerous exceptions have been reported recently [87] and shown in this study. Regarding pMTs1-3, there is no clear picture in what tissues, at what developmental stages, and in response to which stimuli they are expressed. The observed up-regulation of *BnMT4* and down-regulation of *BnMT1-3* in response to NaCl and *S. plymuthica* supports the hypothesis about the specific role of each type of pMT. The observed up-regulation of *BnMT4* might be related to increased ABA content in *B. napus* seedlings in response to salt stress. The significant up-regulation of this type of pMT specifically by ABA was demonstrated previously, e.g., in germinating wheat seeds [88], in developing wheat pollen embryoids [89], and in *A. thaliana* siliques [90]. Our results also showed that ABA level correlates positively with *BnMT4* expression, further supporting the hypothesis of ABA-mediated regulation of *BnMT4* expression in response to salt stress. On the other hand, *BnMT4* expression negatively correlates with the level of IAA, and the down-regulation of *AtMT4b* by IAA was observed in *A. thaliana* siliques [90]. Moreover, ABA content correlates negatively with *BnMT1* expression, whereas JA positively correlates with *BnMT1* expression. *B. rapa MT2* expression was down-regulated by ABA, SA, and JAMe [79]. Also, in sugarcane (*Saccharum officinarum* L.) seedlings, the expression level of type 2 metallothionein, *ScMT10*, was down-regulated by ABA and JAMe but up-regulated by SA [15]. These observations indicate that plant hormones regulate individual types of plant MTs differently and perform type-specific functions in developmental and stress responses.

## 5. Conclusions

This study showed that salt-tolerant strain *S. plymuthica* originating from polluted areas can increase canola growth in a saline environment. This effect could be mediated by changes in the hormonal balance and the expression of metallothioneins. The results obtained in this study support the hypothesis that each type of MT fulfils specific functions and demonstrate the potential role of type 4 plant metallothionein in salt stress response mediated by ABA.

## Figures and Tables

**Table 1:** List of primers used for qRT-PCR.

Primer Name	Sequence 5′-3′	PCR Product Size (bp)
BnMT1_for	TGGTTCCGCTTGCAAATGTG	90
BnMT1_rev	CTACAGTTTGACCCGCAGCT	
BnMT2_for	CTGTGGTTGTGGATCTGGCT	118
BnMT2_rev	TGCAACGCCGAAGACAAAAG	
BnMT3_for	AGACCCAGTGCGTGAAGAAG	110
BnMT3_rev	CCCGTTCTCTTCTGCACCAT	
BnMT4_for	AGGCAAAGGAACCTCAGTCG	112
BnMT4_rev	TTGATCCCCACCAGATGCTG	
BnPP2A_for	AGGGCTATCACCTTCTC	85
BnPP2A_rev	ACACATTGGTCCTTCGT	
BnRPL_for	CACACTCACCACCGCAAGGGC	143
BnRPL_rev	GGATGACGGAAGGCGACGCG	
BnSAND_for	ATACCGAGCATACCAGAA	108
BnSAND_rev	GTGACCCAGCATAGCAGA	

## Data Availability

The raw data supporting the conclusions of this article will be made available by the authors upon request.

## Supplementary Materials

The following supporting information can be downloaded at: https://www.mdpi.com/article/10.3390/genes16020166/s1, Table S1: The numbers and function of putative *cis*-regulatory elements identified in promoter regions of *BnMT1-4* gens using PlantCARE database.

## Abbreviations

The following abbreviations are used in this manuscript:

ABA	abscisic acid
CRE	cis-regulatory element
GA	gibberellins
IAA	indole-3-acetic acid
JA	jasmonic acid
MeJA	methyl jasmonate
MT	metallothioneins
PGPR	plant growth-promoting rhizobacteria
SA	salicylic acid
